# Spatiotemporal network structure among “friends of friends” reveals contagious disease process

**DOI:** 10.1371/journal.pone.0237168

**Published:** 2020-08-06

**Authors:** Carmel Witte, Laura L. Hungerford, Bruce A. Rideout, Rebecca Papendick, James H. Fowler

**Affiliations:** 1 Disease Investigations, San Diego Zoo Global, San Diego, California, United States of America; 2 Department of Family Medicine and Public Health, University of California, La Jolla, California, United States of America; 3 Graduate School of Public Health, San Diego State University, San Diego, California, United States of America; 4 Department of Population Health Sciences, Virginia-Maryland College of Veterinary Medicine, Blacksburg, Virginia, United States of America; 5 Department of Political Science, University of California, San Diego, La Jolla, California, United States of America; 6 Division of Global Public Health, University of California, San Diego, La Jolla, California, United States of America; Georgia State University, UNITED STATES

## Abstract

Disease transmission can be identified in a social network from the structural patterns of contact. However, it is difficult to separate contagious processes from those driven by homophily, and multiple pathways of transmission or inexact information on the timing of infection can obscure the detection of true transmission events. Here, we analyze the dynamic social network of a large, and near-complete population of 16,430 zoo birds tracked daily over 22 years to test a novel “friends-of-friends” strategy for detecting contagion in a social network. The results show that cases of avian mycobacteriosis were significantly clustered among pairs of birds that had been in direct contact. However, since these clusters might result due to correlated traits or a shared environment, we also analyzed pairs of birds that had never been in direct contact but were indirectly connected in the network via other birds. The disease was also significantly clustered among these friends of friends and a reverse-time placebo test shows that homophily could not be causing the clustering. These results provide empirical evidence that at least some avian mycobacteriosis infections are transmitted between birds, and provide new methods for detecting contagious processes in large-scale global network structures with indirect contacts, even when transmission pathways, timing of cases, or etiologic agents are unknown.

## Introduction

Avian mycobacteriosis is a bacterial disease that has long been considered contagious, passing indirectly between birds through the fecal-oral route [1,2]. However, recent long-term studies in well-characterized cohorts have found low probabilities of disease acquisition among exposed birds [3,4] and multiple strains and species of mycobacteria associated with single outbreaks [5–8]. These findings suggest pre-existing environmental reservoirs of potentially pathogenic mycobacteria may be a cause of many avian infections, as is the case with non-tuberculous mycobacterial (NTM) infections in humans and other animals [9–11]. Both transmission pathways are biologically plausible, but differentiating between the two in real world populations is very difficult: exposure to environments where mycobacteria could have been indirectly transmitted from another bird is the same as exposure to environments where the infection could have been acquired from an exclusively environmental source.

Social network analysis has been widely used to study the transmission of phenomena between individuals [12–14]. The first step in these analyses is usually to measure the extent to which incidence is correlated between connected individuals in the network [15]. However, there are multiple reasons why disease may cluster on a network, besides direct or indirect transmission. Exposure to a source or a vector in the shared environment may lead to spatial or temporal clustering of cases [16–18]. Homophily, or the tendency for connected individuals to have similar characteristics, [19] can also result in clustering of cases within networks. In addition, the presence of multiple pathways of infection acquisition, unknown etiologic agents, or inexact information on the timing of infection make the detection of true transmission events difficult. Importantly, all of these processes may work simultaneously to obscure transmission pathways.

Here, we develop a novel “friends-of-friends” method to infer whether disease is being transmitted through a social network. Specifically, we hypothesize that detectable, global network patterns of disease can be differentiated by comparing correlations between directly connected individuals (“friends”) and between indirectly connected individuals (“friends of friends”). When disease is caused by transmission, birds that are directly exposed to one another will exhibit correlation in incidence. Likewise, as the disease spreads from bird to bird, it will create correlation in incidence between indirectly connected individuals as well [15]. These indirect correlations will also be time-dependent, creating “directionality” in the network [12,15]. We expect future incidence to depend on past exposure, and not the other way around.

Infections acquired from an exclusively environmental source of mycobacteria may also exhibit correlations between directly connected birds because they share the same environment. This would be analogous to clusters of cases of NTM infections in humans arising from common exposure to environments that harbor mycobacteria, such as soil [20] or heating and cooling devices in hospitals [16,21]. However, environmental sources cannot explain correlations in incidence between birds that share exposure to a common bird but not to a common environment.

Among humans or animals in the wild it is difficult to observe whether friends of friends have ever shared the same environment. For example, many pairs of indirectly connected individuals in the Framingham Heart Study social network live in the same small town [12,13]. In wildlife it takes extensive trapping, radio tracking, or proximity loggers to infer space-sharing and contact [22–26]. However, in zoo populations individuals are typically assigned to specific enclosures. If animals are sometimes transferred between these enclosures, it creates a directed network of exposure in which some individuals may be indirectly exposed to other individuals but not to a common environment. For example, if bird C is moved from an enclosure with bird A to an enclosure with bird B, then B is exposed to A via C, even though they never shared an enclosure in common. And, importantly, since disease transmission is time dependent, B is exposed to A, but A is not exposed to B. This allows a further reverse-time placebo test of whether or not correlations in the network are due to transmission. Our friends-of-friends method has broad applicability for assessing global patterns of transmissibility when the network is complete and connectivity well-defined, but the disease etiology or transmission pathways are unknown.

## Methods

### Source and study population

San Diego Zoo Global houses one of the largest, breeding bird populations in the world, historically averaging over 3,000 birds at any given time across two facilities, the San Diego Zoo and San Diego Zoo Safari Park (collectively referred to as San Diego Zoo Global, SDZG). Birds are frequently moved among enclosures for breeding, behavior or other management reasons, as well as imported from or exported to other institutions. This creates a dynamic network of contacts over time that varies individual exposure to environments and other birds.

The source population included 16,837 birds present at SDZG between 1 January 1992–1 June 2014 that were at least 6 months old and present for at least seven days. All birds in this population were under close keeper observation and veterinary care during the entire study period and received complete post-mortem exams if they died. Birds in this population had documented dates of hatch, acquisition, removal, and death. We excluded a small number of birds (n = 437) because they had incomplete information on enclosure histories. The 16,430 remaining birds had near-complete enclosure tracking over time with move-in and move-out dates for each occupied enclosure. All management data were stored in an electronic database. Thus, the population represents a group of birds for which 1) a near-complete social network could be assembled from housing records that tracked dynamic movement over time, and 2) avian mycobacteriosis disease status could be determined for any bird that died.

All historic data in these retrospective analyses were originally collected for medical activities and animal management purposes unrelated to the present study. For these reasons, the San Diego Zoo Global Institutional Animal Care and Use Committee exempted our study from review for the ethical use of animals in research.

### Identifying cases of mycobacteriosis

If a bird in the source population died, a board-certified veterinary pathologist conducted a thorough post-mortem exam that included histopathology on complete sets of tissues, unless advanced autolysis precluded evaluation. If lesions suggestive of avian mycobacteriosis were observed, then special stains (Ziehl-Neelsen or Fite-Faraco) were used to confirm presence of acid-fast bacilli. Occasionally, clinical presentation permitted antemortem diagnosis based on tissue biopsy. For this study, any bird with acid-fast bacilli present in tissues was considered positive for avian mycobacteriosis at the date of diagnosis.

Birds were classified as ‘infected’ on their date of diagnosis or ‘uninfected’ on their date of death if the post-mortem examination showed no evidence of disease. Birds were also classified as ‘uninfected’ on their date of export if they were still apparently healthy. Birds that were still alive on the study end date of 6/1/2014, were followed for up to the assumed minimum incubation period (further described below; e.g., six months or through 11/28/2014) to determine final disease status.

### Definition of network nodes and edges

The network was defined based on the subset of birds that qualified as subjects and their friends (network nodes), and the connections between them (network edges). Study subjects included all birds from the source population with complete information on history of exposure to other birds. This included both birds that hatched in the population, as well as birds imported from elsewhere. If a bird was imported, then it must have been present for a duration equal to or greater than the maximum incubation period (further defined below); those that were present for less time were not included as a study subject because they could have been infected prior to importation.

**Fig 1:**
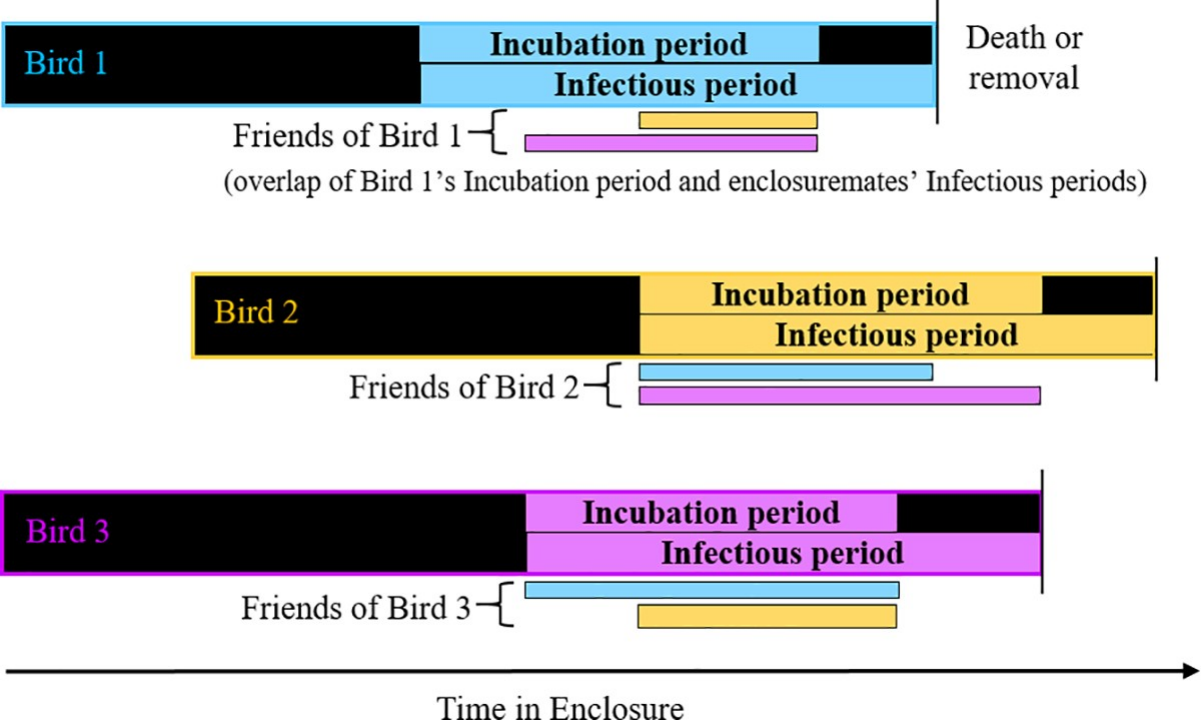
Diagram of potential transmission relationships and connectivity of birds in the network. The figure represents three example birds, assessed for the potential for each to have acquired infection from the other. Each bird, or “subject”, was defined to have an incubation period, initially set to the period occurring six to 24 months before the bird’s final date in the study. Any other bird that shared an enclosure with the subject during its incubation period was defined as a “friend” if the two birds shared the space during the second bird’s infectious period. A friend’s infectious period was initially set to the period occurring two years prior to its final date in the study. Thus, the figure shows the incubation and infectious periods for each bird in the larger bars while the smaller bars show the overlapping period when the other two birds would be defined as its infectious”friends”. The network edges were created from identifying the spatial and temporal overlap of potential incubation and infectious periods of subjects and friends in the study population.

Any bird that directly shared an enclosure with a subject for at least seven days was considered a “friend”. Thus, the same bird could serve as both a subject as well as a friend for other birds, as illustrated in Fig 1. Spatial connections between subjects and friends were determined through cross-referencing enclosure move-in and move-out dates of all birds. Contact occurring in a few enclosures, including hospital and quarantine enclosures, could not be determined and was therefore excluded.

Exposures that could lead to potential transmission of mycobacteriosis would be those which occurred within the incubation period of the subject (Fig 1). However, the distribution of the true incubation period for avian mycobacteriosis is unknown. As a starting point, minimum incubation period, i.e., the minimum time for an exposure to result in detectable disease, was set to six months. This was based on early literature from experimental studies that mimicked natural transmission [27,28]. This is also consistent with our own data where the earliest case in the population occurred at 182 days of age [3]. The maximum incubation period was set to two years. Early studies reported deaths occurring up to 12–14 months after infection [27–29]; however, some authors reviewed by Feldman [1] considered it possible that the disease progression could take years. For subjects that were classified as non-infected, this same interval (two years to six months prior to death or censoring) was used to identify contact with friends. For example, if a subject died on January 1, 2005, it would be connected to all friends with which it shared an enclosure for at least seven days within the time window of two years until six months prior to the subject’s death, or between January 1, 2003 and July 1, 2004.

**Fig 2:**
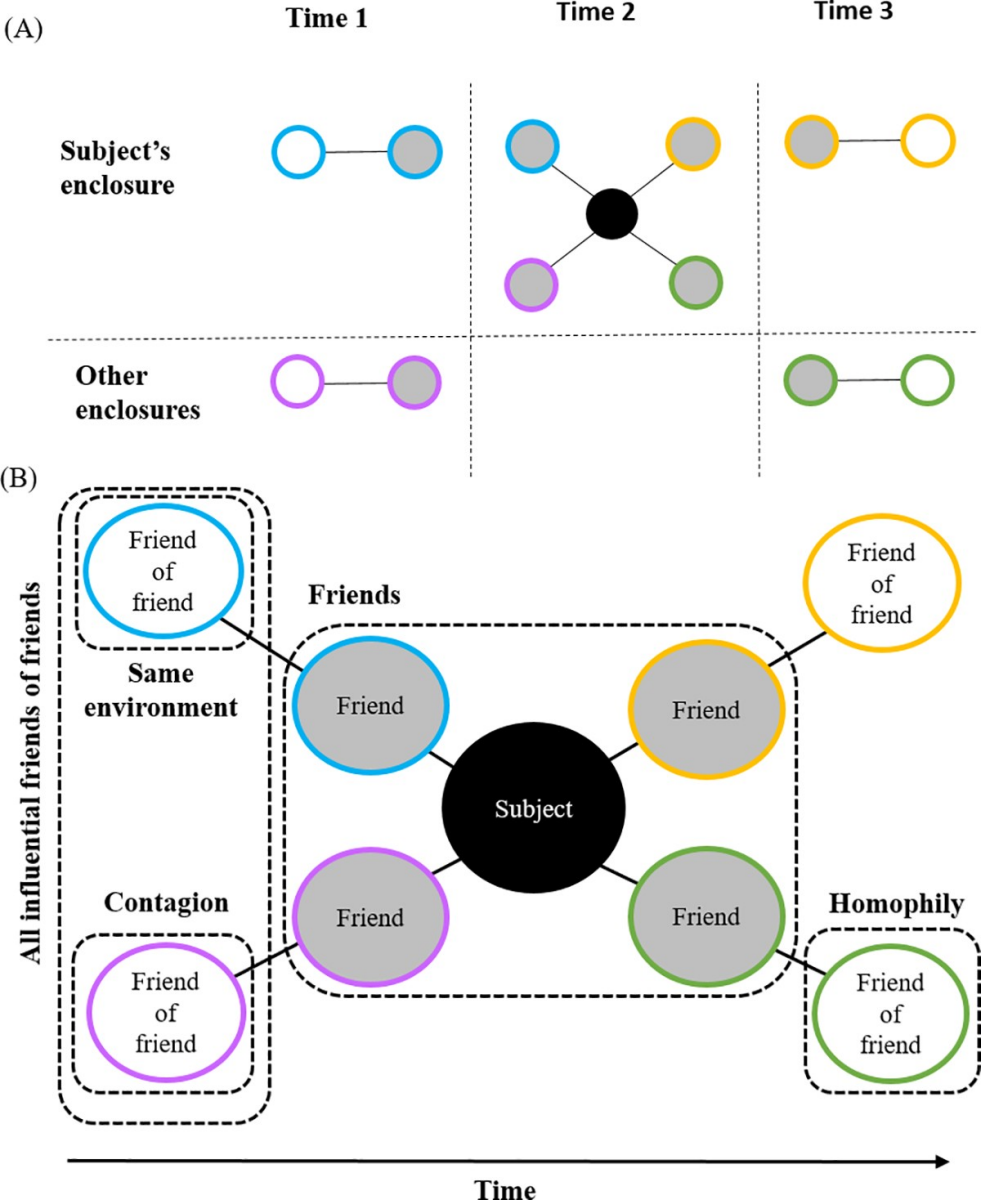
Illustration of network assembly and evaluated relationships. Each circle represents a different bird, showing a subject (black), their friends (gray), and their friends of friends (white). Colors represent pathways along which the different evaluated relationships were formed. Lines represent the network edges which connect birds that shared enclosures. **(A)** Illustration of network construction. Friends of friends differed by the timing of contact (those that could vs. could not transmit infection to the subject) and location of contact (same enclosure vs. different enclosure) with respect to their subject. **(B)** The subject’s assembled network of friends, friends of friends, and evaluated relationships. *Friends*: Clustering of disease associated with direct contacts. *Friends-of-Friends*: *All influential*. Clustering of disease associated with all indirect contacts that could influence the subject’s disease status (based on timing of contact). *Friends-of-Friends*: *Same environment*. Clustering of disease associated with influential indirect contacts that were exposed to the same enclosure/environment. *Friends-of-Friends*: *Contagion*. Clustering of disease associated with influential indirect contacts that were never exposed to the same environment. This evaluation is key for removing the confounding effects of the environment and testing for a contagious process. *Friends-of-friends*: *Homophily*. Clustering of disease associated with friends of friends that were never exposed to the same environment and could not have transmitted disease to the subject based on the timing of the connection. This reverse-time placebo test evaluates our data for the presence of homophily, or whether disease clustering can be explained by similarities among connected birds.

Exposures of subjects to friends that could lead to potential disease transmission would also be those which occurred within the friends’ infectious periods when the bacteria could spread to other birds (Fig 1). The period of shedding during which a bird is infectious for other birds is unknown and no estimates were available for a naturally occurring disease course. Therefore, friends were assumed to be infectious for the maximum incubation time, or two years, as a starting point. Exposure of the subject to friends that were not infected was considered for the same two-year period prior to the friend’s final date in the study. Fig 2A and 2B illustrates network assembly over time for an example subject and its friends.

### “Friends-of-Friends” network model

The transmission network was graphed using the Kamada-Kawai [30] algorithm and all visualizations and analyses were performed using R software, package: igraph [31]. An initial network was structured to include all connections of seven days or more between birds that occurred during their lifetimes. From this, the transmission network used in the analyses was constructed by refining connectivity based on the subjects’ incubation periods and friends’ infectious periods as described above. Network topology was characterized by size (number of nodes and edges), average path length, and transitivity (probability that two connected birds both share a connection with another bird).

To evaluate statistically whether or not disease status of a subject is predicted by the disease status of its friend, we calculated the probability of mycobacteriosis in a subject given exposure to an additional infected friend relative to the probability of mycobacteriosis in a subject exposed to an additional non-infected friend, i.e., the relative risk (RR). To determine significance of the RR, the observed RR was compared to the distribution of the same RR calculation on 1000 randomly generated null networks where the network topology and disease prevalence were preserved, but the disease status was randomly shuffled to different nodes [15,32]. If the observed RR fell outside the range of permuted values between the 2.5^th^ and 97.5^th^ percentiles, i.e., the null 95% confidence interval (CI), then we rejected the null hypothesis that the observed relationship was due to chance alone. Reported p-values were estimated from the null 95% CI.

We evaluated the relative risk of disease transmission through five types of shared relationships between subjects and their friends (Fig 2B). Each evaluation targeted different groups of subject-friend pairs that varied in degrees of separation as well as spatial and temporal characteristics of network edges.

### Friends

Risk (also referred to as “clustering”) of disease associated with directly connected birds, or “friends”. This analysis examined all pairs of birds where the subject was in direct contact with its friend during the subject’s defined incubation period and the friend’s infectious period (illustrated in Fig 1). The RR estimate includes the combined risk from direct exposure to both other infected birds and a common environmental source.

### All influential friends-of-friends

This analysis examined whether associations persisted among the indirectly connected friends, as observed in other contagious processes [15]. To identify these friends of friends, we constructed a matrix of shortest paths between all subject-friend pairs that never directly shared an enclosure but were indirectly connected through an intermediary bird. Before estimating the RR and conducting the random permutation tests, the data were limited to each subject’s set of “influential” nodes, or the friends of friends connected by pathways that respect time ordering along which disease could propagate [33]. In other words, the friend of friend shared an enclosure with an intermediary bird *before* the intermediary bird contacted the subject. The estimated RR includes the indirect risk of disease from both contagion and exposure to a common environmental source.

### Friends-of-friends: Same environment

Risk of disease transmission associated with influential friends of friends sharing an environment with their subject. This analysis examined associations with the subset of all influential friends of friends, where both birds were in the same enclosure but not at the same time. For example, bird A shares an enclosure with C. If A moves out and B subsequently moves in, then B is exposed to A via C. Importantly, both A and B also were exposed to the same environment. Associations in this group would reflect a combination of risk due to common environmental exposure and contagion.

### Friends-of-friends: Contagion

For contagion, we evaluated associations with the influential friends of friends that were never in the same enclosure as their subject. From the earlier example, if bird C is moved from an enclosure with bird A to an enclosure with bird B, then B is exposed to A via C. That is, A can transmit infection to B, even though they never shared an enclosure. Case clustering could not be attributed to exposure to the same environment because the subject and its friends of friends were never housed in the same enclosure. This evaluation also ensured correct temporal alignment between exposure to an infectious agent and disease outcome in the subject. This comparison was key for removing confounding effects of environmental exposure and testing for a contagious process.

### Friends-of-friends: Homophily

Although disease clustering among friends of friends could represent a contagious process, there is a possibility that some of the association could be explained by homophily, i.e., that connected birds could be more alike than the general bird population in terms of species, behavior, susceptibility, enclosure characteristics, etc. [19]. This could make both birds more likely to acquire infection from any source and manifest as clustering on a network at degrees of separation.

We tested the network for the presence of homophily using a reverse-time placebo test. For this test, we evaluated disease clustering between a subject and its friends of friends from different enclosures that could not have transmitted infection based on the timing of the contact. For the tests of contagion, we described how B could be exposed to A via C; however, in that same example, the reverse would not be true. B could not transmit infection to A because disease transmission is time-dependent. For our reverse-time placebo test, we evaluated whether the infection status of B predicted the infection status in A. If so, then it would suggest homophily is present and driving disease clustering.

### Sensitivity analyses

Sensitivity analyses were performed to compare differences in RR estimates while varying model assumptions. We varied subjects’ incubation time (testing a minimum of three months and a maximum of one, three, four and five years) and friends’ infectious time (two years, one year, and six months). We also refined network edges to evaluate associations in subsets of data where biases were minimized. This included limiting the friends to those whose exposure to the subject was exclusively outside of the two-year infectious window. It also included refining network edges to contact between subjects and friends that occurred only in small enclosures where enclosure sharing may be a better proxy of true exposure. Finally, we limited analyses to subjects and friends that died and received a post-mortem examination.

## Results

The 16,430 birds in the source population consisted of 950 species and subspecies housed across 848 enclosures. Mycobacteriosis was diagnosed in 275 of these birds (1.7%). The subset that qualified as study subjects included 13,409 of the birds, which represented 810 species and subspecies. Subjects were housed across 837 different enclosures that varied in size, housing anywhere from one to over 200 birds at any given time. In total, 203 (1.5%) subjects developed mycobacteriosis. Subjects were present in the study population for variable amounts of time with the median follow-up being 3.4 years (IQR: 1.4–7 years). On average, subjects moved between enclosures 4.4 times (SD: 4.1; range: 0–71), and were housed in three separate enclosures (SD: 2.5; range 1–26). The average time a subject spent with each friend was about ten months (314 days; SD: 201 days).

**Fig 3:**
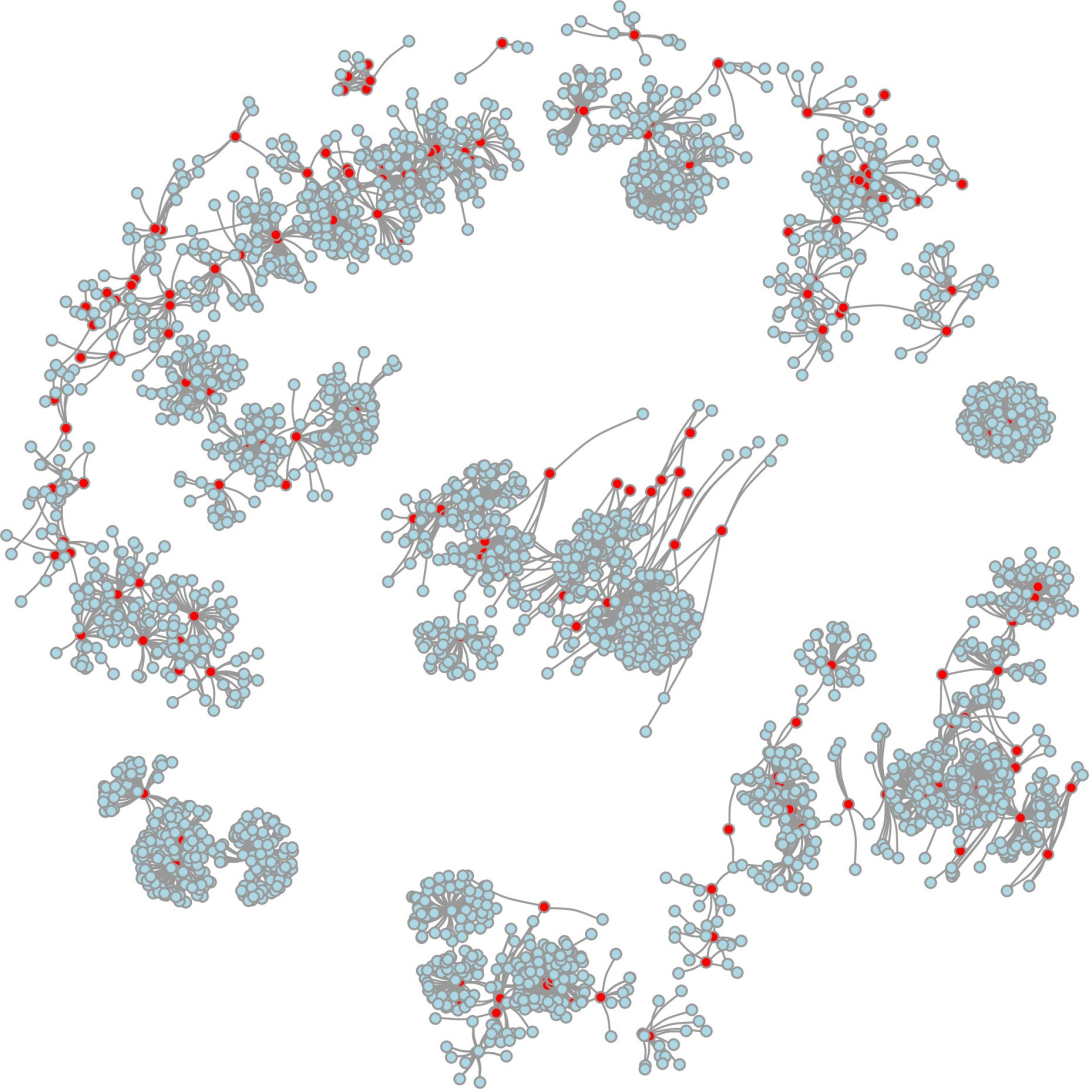
Social network graph of a subset the San Diego Zoo Global bird network, 1992–2014. The subset of the network illustrates all positive study birds (“subjects”) and their direct contacts (“friends”). Each node represents one bird in the data set and connections between birds were defined by enclosure sharing. There are 3417 birds represented in this subset with 6066 unique connections between them. The color of the circle indicates each bird’s disease status: red denotes a bird with mycobacteriosis and light blue denotes a bird that did not have disease. Statistical tests for clustering of disease on the network showed significant increases in disease risk for subjects directly or indirectly connected to an infected friend, compared to an uninfected friend.

The full network that included all subject-friend connections contained 2,492,438 edges, but we focused on the transmission network limited to plausible fecal-oral transmission routes based on sharing an enclosure for at least 7 days during the subjects’ incubation periods and its friends’ infectious periods. This transmission network included all 16,430 birds with 905,499 connections linking their temporal and spatial location. The median number of friends each subject contacted (network degree centrality), was 105 (IQR: 21–303; range: 0–1435). The network exhibited small world properties [34] with short paths (average path length = 3.8) and many cliques where groups of birds were all connected to each other (transitivity = 0.63). A portion of the network diagram that includes subjects infected with avian mycobacteriosis and their directly connected friends is shown (Fig 3).

**Fig 4:**
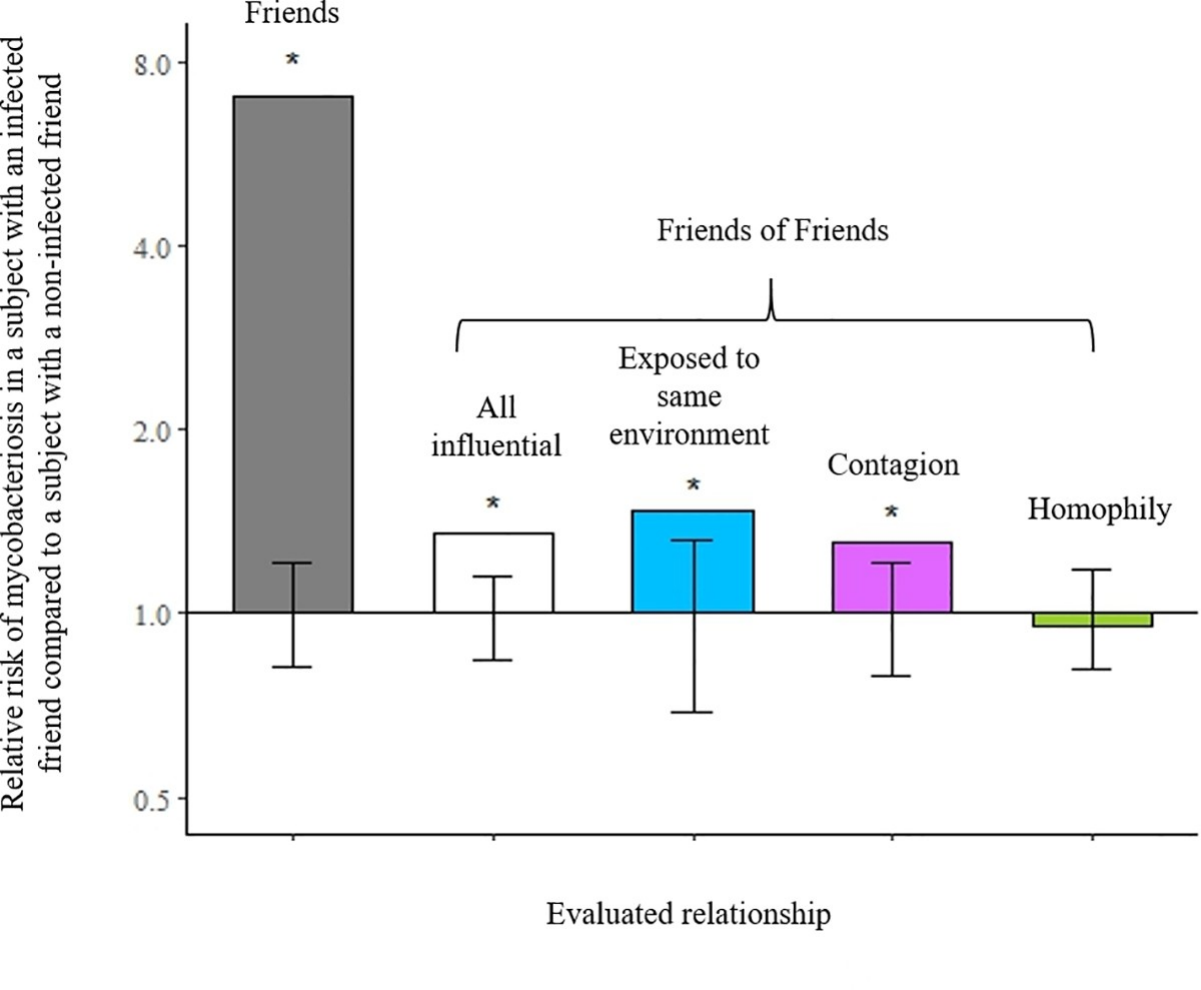
Estimates of disease clustering between friends in the bird transmission network, San Diego Zoo Global (n = 16,430). The estimated relative risk (RR) for each of five different relationships between subjects and friends that were directly and indirectly connected. Evaluated relationships are described in the Methods and Fig 2B. Significance of the estimate was determined by comparing conditional probability of mycobacteriosis in the observed network with 1000 permutations of an identical network (with the topology and incidence of mycobacteriosis preserved) in which the same number of infected birds were randomly distributed. Error bars show the null 95% confidence intervals generated from the random permutations. RRs that were outside of the null and significant are indicated with *.

Results from all five associations are shown in Fig 4 and RR estimates with p-values are reported in Table 1. When we performed our test between the subject and its directly connected friends we found significant clustering of cases based on social network ties; the risk of mycobacteriosis given exposure to an infected friend was 7.0 times greater than the risk of mycobacteriosis given exposure to an uninfected friend (p<0.001).

**Table 1:** Results of Friends-of-friends network analysis and sensitivity analyses for birds housed at San Diego Zoo Global, 1992–2014 (n = 16,430).

			Relative risk and p-value^a^				
Analysis	Subjects	Network Edges	Friends	Friends of friends: All influential	Friends of friends: Same environment	Friends of friends: Contagion	Friends of friends: Homophily
**Transmission network**	** **	** **	** **	** **	** **	** **	** **
Subject incubation period: min 180 days, max 2 yrs; Friend infectious period: 2 yrs	13,409	905,499	7.00 p<0.001	1.35 p<0.001	1.47 p = 0.004	1.31 p = 0.004	1.18 p = 0.586
**Sensitivity analyses**							
Modified subject incubation: min, max							
90 days, 2 yrs	13,402	969,958	7.03 p<0.001	1.44 p<0.001	1.38 p<0.001	1.45 p<0.001	1.05 p = 0.586
180 days, 1 yr	14,655	693,347	7.24 p<0.001	0.97 p = 0.779	1.54 p = 0.010	0.77 p = 0.071	0.78 p = 0.097
180 days, 3 yrs	12,567	1,041,593	4.35 p<0.001	1.35 p<0.001	1.29 p = 0.022	1.36 p<0.001	1.08 p = 0.263
180 days, 4 yrs	11,924	1,142,294	3.43 p<0.001	1.34 p<0.001	1.62 p<0.001	1.27 p<0.001	1.03 p = 0.674
180 days, 5 yrs	11,352	1,227,119	2.99 p<0.001	1.3 p<0.001	1.6 p<0.001	1.23 p<0.001	1.02 p = 0.763
Modified friend infectious period							
180 days	13,409	542,181	7.31 p<0.001	1.23 p = 0.040	1.47 p = 0.013	1.13 p = 0.327	0.87 p = 0.308
1 yr	13,409	707,014	7.62 p<0.001	1.25 p = 0.006	1.45 p = 0.003	1.18 p = 0.142	0.86 p = 0.19
Birds with post- mortem data	5,369	905,499	3.3 p<0.001	1.24 p<0.001	1.46 p<0.001	1.16 p = 0.002	0.96 p = 0.476
Only birds in small enclosures	11,069	204,847	5.19 p<0.001	1.39 p = 0.005	1.47 p = 0.065	1.35 p = 0.044	1.07 p = 0.738
Contact before the friend’s infectious period (>2 years prior to removal or death of friend)	13,409	604,078	1.54 p<0.001	1.12 p<0.001	1.21 p<0.001	1.08 p = 0.025	1.10 p = 0.014

RR = Relative Risk; CI = confidence interval. Significant associations (p<0.05) are shown with a gray background.

^a^The five evaluated relationships are described in detail in the Methods and Fig 2B. The calculated statistic is the probability that a subject has disease, given that its friend has disease, compared to the probability that a subject has disease given that its friend does not (i.e., RR). To determine whether the observed RR falls within the 2.5^th^ and 97.5^th^ percentile of the null distribution, the disease status was randomly shuffled in 1000 network permutations where the network structure and prevalence of mycobacteriosis was preserved. Significant p values indicate that the observed RR fell outside of the null 95% CI and we reject the null hypothesis that the observed RR is due to chance alone.

Significant associations persisted among the friends of friends. The RR of disease given exposure to any influential, infected friend of friend, compared to exposure to an uninfected friend of friend, was 1.35 (p<0.001). When subset to just the influential friends of friends that shared the same environment, the RR was 1.47 (p = 0.004). Importantly, the friends-of-friends contagion model identified a significant 31% increase in risk of infection among subjects that were exposed to an infected friend of friend compared to those exposed to an uninfected friend of friend (RR: 1.31, p = 0.004). We found no evidence of homophily with our reverse-time placebo test; i.e., there was no significant association when the friends of friends were limited to those who may have correlated traits, but could not have influenced the subject’s disease status based on location and timing of their indirect connection (RR: 0.95; p = 0.586).

Results of sensitivity analyses for all five evaluated relationships are shown in Table 1. The sensitivity analyses did not yield drastically different findings than the analyses of the main network and the significance of most associations remained. Generally, as the subjects’ incubation periods increased, the magnitude of the RRs with the friends and friends of friends decreased. This same pattern was observed when connectivity was limited to that occurring two years prior to the friends’ removal dates (i.e., outside of the friends’ incubation windows). Patterns of significance were mostly unchanged when the network was limited to just animals with post-mortem exams, and just birds housed in small enclosures. Importantly, significant disease clustering in the test for contagion persisted in most examined network variations. The exception to this is when the subjects’ maximum incubation periods or the friends’ infectious periods became more narrowly defined. Homophily was detected only when network edges were restricted to exposures outside friends’ incubation periods when long time spans were present (RR: 1.10; p = 0.014).

## Discussion

Our friends-of-friends network analysis suggests that avian mycobacteriosis can spread through bird social networks. Although connected birds may acquire infection from exposure to common environmental sources and may share features that make them more likely to acquire disease through the environment, our friends-of-friends method detected statistically significant bird-to-bird transmission.

One of the biggest challenges in determining if bird-to-bird contagion is present for infectious agents that are present in the environment, such as mycobacteria, is distinguishing the role of the environment. In one scenario, the environment serves as an intermediate collection place for mycobacteria being passed via (mostly) fecal contamination from an infected bird to one or more other birds, leading to infection spread in chain- or web-like patterns across a network [35]. Alternatively, the environment may serve as the natural, independent reservoir of mycobacteria (e.g., biofilms in the water [36]) giving rise to opportunistic infection among birds that share the location. Spatial and temporal disease clustering could represent either or both of these two infection routes. Homophily, where connected individuals tend to be more alike in species or habitat needs than the general population, and, therefore, may share the same disease susceptibility, could occur in both scenarios. For the directly connected birds in our study, the significantly elevated RR represented a combination of these three effects.

Examining the friends of friends rather than directly connected birds provided a means to disassociate exposure to another bird from exposure to that bird’s environment. At two degrees of separation, the characteristics of network edges were more distinct, with temporal separation in potential transmission pathways and spatial separation in location. We exploited these pathways in a stepwise approach to calculate the RR of disease given exposure to friends of friends with different types of network ties. The subset of all influential friends of friends were temporally aligned to pass infection, but this group again represented a combined effect of multiple transmission pathways. Because there was no evidence of significant homophily (further discussed below), we could use the network structure to test for the presence of contagion. Among subjects who were connected to infected friends of friends in a different enclosure, the significant increase in risk for mycobacteriosis represents contagion. While this very specific subset of network edges allowed us to disentangle environmental and contagious transmission, it required two consecutive infections among a chain of related birds. This ignored most subjects and their friends of friends that shared enclosures where both processes were possible and completely confounded. While our extensive, long-term set of connections in this network allowed detection of disease transmission using just this subset, the relative risks likely underestimate the true magnitude of bird-to-bird contagion.

Our data show significant, directional clustering along the pathways on which disease could propagate; however we did not find clustering when we reversed these pathways—where birds were connected, but disease could not be transmitted because passing an infection cannot move backwards through time. We applied our test of directionality, which is similar to those used by others [15], to evaluate whether homophily could be driving the observed associations. In this bird population, similar species with comparable habitat needs have always tended to be housed together. Therefore, we would expect biases due to homophily would exert similar effects along all pathways of connectivity, regardless of time. It is well documented that homophily and contagion are confounded in social networks [37,38] and we could not specifically adjust the RRs for unobserved homophily; however many of the psychosocial factors that lead to homophily in human networks [19,38] are not directly applicable to birds. While homophily might still be present, our data strongly suggest that it is not driving the observed clustering of disease between a subject and its friends of friends.

Historically, in experimental infection studies, birds have been shown to be susceptible to the infectious bacilli when directly administered, i.e., introduced intravenously, intramuscularly, intraperitoneally, subcutaneously, or orally [39–42]. Yet, the relevance of direct inoculation to natural transmission has always been tenuous. Some studies have shown little to no transmission when healthy chickens were placed in contact with either diseased birds or their contaminated environments [43]. Therefore, our study provides new evidence, which supports bird-to-bird transmission in natural settings. Our results also suggest that avian mycobacteriosis is not highly contagious, which is consistent with early experimental studies that conclude the bacteria must be given repeatedly over long periods of time to ensure infection [1]. The small world network structure that we identified for birds in the study population would predict epidemic-style outbreaks for diseases with facile and rapid transmission [34,44]; however, most birds did not acquire infection even when directly linked to other positive birds for long periods. Over time, we have not seen epidemics and the incidence of disease in this population is consistently low (1%) [3]. Our network approach was elucidating in this particular scenario, enabling us to uncover subtle patterns of a contagious process.

Environmental mycobacteria are recognized as the cause for NTM infections in humans and other animals [9–11]. Limited genetic and speciation data from managed avian populations have found multiple strains and species of mycobacteria attributed to single outbreaks [6–8]. In our bird population, several different species and genotypes of mycobacteria have also been identified [5,45]. Consequently, we know that some birds could not have passed the infection to each other. Genetic data from mycobacterial isolates would be a more definitive method of identifying the transmission of infection within a shared environment. For the present study, our approach was to isolate and test for contagion when there is missing information on the specific etiologic agents and transmission pathways. Additional studies using genetic data could refine relevant transmission pathways or highlight important environmental sources within the network.

We took care in assembling our network to ensure that the edge construction between subjects and friends adhered to general recommendations for disease networks [26,46,47]. This included incorporating biologically meaningful time-periods relevant to mycobacterial disease ecology and the type of exposure needed for transmission. Generally, mycobacteriosis is considered a chronic disease, with an incubation period that can last for months and possibly years [1,2]. It is also thought that animals can insidiously shed the organisms for long periods of time and those organisms can potential stay viable in the environment for years [48,49]. We know there is misclassification of exposure in this network, because the true extents of incubation and infectious periods are wide, variable, and unknown. In sensitivity analyses, our RR estimates were generally similar when we varied incubation and infectious periods (Table 1). We did find a significant RR when limiting network edges to those occurring before the friends’ 2-year incubation period, which suggests that some contagious processes may occur before the 2-year window. We also found that evidence for contagion was lost when either the subject incubation period or friend infectious period was short (less than six months and less than one year, respectively). It is likely that the shorter incubation times did not allow sufficient overlap of risk periods between subjects and friends.

The duration of exposure needed for transmission is also unknown, but birds can be housed together for a year or more and not acquire infection [1,4]. Generally, mathematical models show that increasing the intensity or duration of contact between individuals with an infectious disease increases the probability of a transmission event and this can be reflected in weighted networks [35,50]. In the present study, we required a minimum of seven days together to establish a network link that could capture relevant, short-duration exposure; however, the majority of birds were together for longer, with the mean contact-days being about 10.5 months (314 days). Further exploration of contact heterogeneity on network associations may provide additional insight into clinically relevant exposure, infectious periods, and incubation times.

Inferring contagion by testing for disease clustering in subsets of the network requires quite complete network ascertainment, very good information on location over time, knowledge of disease outcomes, and a large number of subjects and their connected friends over time. Our zoo data were unique in this respect and represent an example of how network substructures can inform global disease processes. Many of the issues that cause bias in network measures, such as node censoring [51], network boundary specification [52], or unfriending [53] are unlikely to have affected our findings due to the completeness of our data. While such data may currently be rare, large datasets with similar network resolution may become widely available in the future as the world becomes increasingly connected by technology. For example, many new public and private contact-tracing initiatives are taking advantage of mobile phone technology to digitally track COVID-19. Eventually, these may allow near-complete human disease transmission networks to be assembled. This makes our friends-of-friends social network approach using network substructures a viable option for informing indirect COVID-19 transmission pathways and public policy.

Most epidemiologic studies that use a network approach focus on directly transmitted, infectious diseases [47]. Social networks to investigate diseases transmitted through the environment are assembled less often because defining contact in the presence of environmental persistence or other important transmission routes, such as fomites or insects, can be challenging [26]. To our knowledge, this is the first application of a friends-of-friends method to determine whether global patterns of connectivity support a contagious process. Similar approaches could be useful to investigate diseases of humans or animals when the network is complete and mobility patterns are known, but the disease etiology or transmission pathways are unknown.
